# Comparative Genome-Wide Transcriptome Analysis of *Brucella suis* and *Brucella microti* Under Acid Stress at pH 4.5: Cold Shock Protein CspA and Dps Are Associated With Acid Resistance of *B. microti*

**DOI:** 10.3389/fmicb.2021.794535

**Published:** 2021-12-13

**Authors:** Jorge A. de la Garza-García, Safia Ouahrani-Bettache, Sébastien Lyonnais, Erika Ornelas-Eusebio, Luca Freddi, Sascha Al Dahouk, Alessandra Occhialini, Stephan Köhler

**Affiliations:** ^1^Institut de Recherche en Infectiologie de Montpellier (IRIM), CNRS, University Montpellier, INSERM, Montpellier, France; ^2^CEMIPAI, CNRS, University Montpellier, Montpellier, France; ^3^Unité des Zoonoses Bactériennes and Unité d’Epidémiologie, Laboratoire de Santé Animale, ANSES, University Paris-Est, Maisons-Alfort, France; ^4^German Federal Institute for Risk Assessment, Berlin, Germany

**Keywords:** *Brucella*, acid stress, cold shock protein, Dps, transcriptome

## Abstract

Brucellae are facultative intracellular coccobacilli causing brucellosis, one of the most widespread bacterial zoonosis affecting wildlife animals, livestock and humans. The genus *Brucella* comprises classical and atypical species, such as *Brucella suis* and *Brucella microti*, respectively. The latter is characterized by increased metabolic activity, fast growth rates, and extreme acid resistance at pH 2.5, suggesting an advantage for environmental survival. In addition, *B. microti* is more acid-tolerant than *B. suis* at the intermediate pH of 4.5. This acid-resistant phenotype of *B. microti* may have major implications for fitness in soil, food products and macrophages. Our study focused on the identification and characterization of acid resistance determinants of *B. suis* and *B. microti* in Gerhardt’s minimal medium at pH 4.5 and 7.0 for 20 min and 2 h by comparative RNA-Seq-based transcriptome analysis, validated by RT-qPCR. Results yielded a common core response in both species with a total of 150 differentially expressed genes, and acidic pH-dependent genes regulated specifically in each species. The identified core response mechanisms comprise proton neutralization or extrusion from the cytosol, participating in maintaining physiological intracellular pH values. Differential expression of 441 genes revealed species-specific mechanisms in *B. microti* with rapid physiological adaptation to acid stress, anticipating potential damage to cellular components and critical energy conditions. Acid stress-induced genes encoding cold shock protein CspA, pseudogene in *B. suis*, and stress protein Dps were associated with survival of *B. microti* at pH 4.5. *B. suis* response with 284 specifically regulated genes suggested increased acid stress-mediated protein misfolding or damaging, triggering the set-up of repair strategies countering the consequences rather than the origin of acid stress and leading to subsequent loss of viability. In conclusion, our work supports the hypothesis that increased acid stress resistance of *B. microti* is based on selective pressure for the maintenance of functionality of critical genes, and on specific differential gene expression, resulting in rapid adaptation.

## Introduction

Brucellae are facultative intracellular coccobacilli causing brucellosis, a widespread bacterial zoonosis, infecting wildlife animals, livestock and humans. Transmission routes of *Brucella* spp. to humans include aerosols, direct contact with infected animals, and most frequently, ingestion of contaminated and unpasteurized dairy products (Pappas et al., 2005). *Brucella abortus*, *Brucella melitensis*, and *Brucella suis* are the most relevant species for human infections (Pappas et al., 2005).

After their uptake by the host cell, brucellae establish a specific intracellular niche, the *Brucella*-containing vacuole (BCV), in which the bacteria survive and replicate (Celli, 2019). This vacuole undergoes endosomal maturation during the early phase of infection, interacting rapidly with early and late endosomes as well as lysosomes, resulting in transient acidification to a pH of 4-4.5 (Porte et al., 1999). This acidification is crucial for intracellular replication and induction of the major virulence factor of *Brucella* spp., the type IV secretion system VirB (O’Callaghan et al., 1999; Porte et al., 1999; Boschiroli et al., 2002; Köhler et al., 2002).

In the past, the protein profile of *B. melitensis* in response to an intermediate acid stress at pH 5.5 has been studied by two-dimensional polyacrylamide gel electrophoresis (Teixeira-Gomes et al., 2000). Two chaperones have been reported to be involved in acid stress resistance: ClpB of *B. suis* (Ekaza et al., 2001) and HdeA, regulated by the RNA-binding protein Hfq (Valderas et al., 2005). The importance of the latter was confirmed by microarray-based transcriptome analysis and by the impact of Hfq-inactivation on stress resistance and intracellular survival of *B. melitensis* (Cui et al., 2013). The transcriptional regulator OtpR was described to be important for tolerance to acid stress in *B. melitensis*, and RNA-Seq whole transcriptome analysis under these conditions showed that OtpR regulates genes mainly involved in bacterial metabolism, but also virulence factors such as *virB* and other transcriptional regulators, predicted to be controlled by OtpR-mediated sRNA expression (Vishnu et al., 2017). More recently, two RNA-Seq transcriptome analyses were performed to study the global gene expression profile of *B. melitensis* 16M in adaptation to pH 4.4: The first study identified a two-component system regulator essential for acid resistance, intramacrophagic and *in vivo* survival of the pathogen (Liu et al., 2016). However, experimental conditions were not clearly described, since both minimal and complex medium were mentioned for acid stress, and bacterial mRNAs were supposedly purified using oligo (dT) beads. The second study focused on the comparison of normal and acid pH gene expression profiles in the 16M wild-type and the Rev1 vaccine strain, providing possible explanations for the attenuated virulence of the latter. In the 16M strain, 773 genes were differentially expressed, encoding predominantly transmembrane transporters, oxido-reductase activities and nucleoside triphosphate biosynthetic processes (Salmon-Divon et al., 2019).

All twelve recognized *Brucella* species, including zoonotic species, share highly conserved genomes and are classified on the basis of their host preference, pathogenicity, and phenotypic and biochemical characteristics. New and atypical species and strains, such as *Brucella microti* isolated from common vole, *Brucella inopinata* from humans (Scholz et al., 2008b,^2010^; Tiller et al., 2010), and strains isolated from non-mammal hosts (Soler-Llorens et al., 2016; Al Dahouk et al., 2017; Eisenberg et al., 2017), have been described over the last 15 years. Most of them, isolated from hitherto unknown wildlife hosts and the environment, are characterized by increased metabolic activity and faster growth rates than the classical species, suggesting an advantage for environmental survival (Al Dahouk et al., 2017) and raising the question whether they may be transmitted from these reservoirs to livestock and humans in brucellosis-free areas.

*Brucella microti* has been isolated in Central Europe from soil and wildlife (Scholz et al., 2008a,^2009^; Ronai et al., 2015). Phylogenetically, this species is closer to those pathogenic for human and livestock than to the group of newly described atypical species/strains comprising *B. inopinata* and strains from Australian rodents (Wattam et al., 2014). Its replication rate in murine and human macrophage cells is higher than that of classical species, and *B. microti* is the first *Brucella* species described to be lethal in mice (Jiménez De Bagüés et al., 2010). This lethal phenotype depends on the type IV secretion system VirB (Hanna et al., 2011) and, as we reported lately, on a smooth LPS with an intact O-polysaccharide (Ouahrani-Bettache et al., 2019). In contrast, at sub-lethal doses, *B. microti* is rapidly cleared from infected mice, never gives rise to chronic infection and confers protection.

New and atypical species, and also those isolated from marine mammals, but not classical ones, are characterized by the presence of two functional acid resistance systems conferring extreme acid resistance *in vitro* at pH 2.5 in the presence of glutamate or glutamine: the glutamate decarboxylase (Gad)- and the glutaminase-dependent system AR2_Q (Damiano et al., 2015; Freddi et al., 2017). In *B. microti*, the Gad system was also shown to play an important role in oral murine infection (Occhialini et al., 2012). In classical species, urease has been described to contribute to acid resistance at very low pH, and most *Brucella* strains show urease activity. Analysis of genome sequences revealed the existence of two urease gene clusters, *ure1* and *ure2*. However, the function of *ure2* is not clear, and *ure1* appears to play the principal role in *in vitro* acid resistance at pH 2.0 and in murine infection *via* the oral route (Bandara et al., 2007; Sangari et al., 2007).

In addition to the observed extreme acid resistance, our previous results had shown that *B. microti* was more acid-tolerant than *B. suis* in a synthetic minimal medium at pH 4.5 (Jiménez De Bagüés et al., 2010), mimicking the acidity encountered by *Brucella* in the host cell phago(lyso)some, or possibly in particular soil environments (Scholz et al., 2008a). The lack of transient intramacrophagic mortality of *B. microti* at 7 h post infection, as opposed to the fate of *B. suis*, might also be linked to its increased acid-resistance (Jiménez De Bagüés et al., 2010). Despite highly conserved genome sequences with an overall identity of 99.8% and not more than 130 genes inactivated in either of the two species but intact in the other (Audic et al., 2009), these two species, which represent an appropriate model for classical versus atypical *Brucella* spp. comparisons, are phenotypically distinct and obviously developed species-specific responses to acid stress. Consequently, the more acid-resistant phenotype of *B. microti* may have major implications for fitness and virulence, both in soil and in the host.

To better understand the molecular mechanisms leading to acid resistance of *B. microti* at pH 4.5, we identified and characterized common and species-specific acid resistance determinants by comparative RNA-Seq-based transcriptome analysis of bacteria exposed to pH 4.5 and 7.0 in minimal medium. Our starting hypothesis was, that the gene expression patterns of the two species may differ under the conditions chosen, and that the observed phenotypic difference in acid resistance between *B. suis* and *B. microti* might be due to specific mutations, and/or to differential gene expression. Our analysis revealed at least two genes of *B. microti* with increased expression under acid stress and associated with better survival in minimal medium at pH 4.5, one of which was inactive in *B. suis* due to frameshift mutation.

## Materials and Methods

### Bacterial Strains and Culture Conditions

*Brucella* suis 1330 (ATCC 23444) and *B. microti* CCM4915, as well as derived mutant strains, were grown in Tryptic Soy broth (TSB) at 37°C under BSL-3 conditions. *Escherichia coli* DH5α, used for cloning and plasmid production, was cultured in Lysogeny Broth (LB). For selection of strains carrying antibiotic resistance genes, kanamycin, ampicillin, and chloramphenicol were added to a final concentration of 50 μg/ml each, when needed.

Growth and survival assays for *B. suis* and *B. microti* strains were performed using Gerhardt’s Minimal Medium (GMM) supplemented with vitamins (Hanna et al., 2013), and with ammonium sulfate (1 g/l) replacing glutamic acid, at the appropriate pH values. For stress survival assays, stationary phase pre-cultures of *Brucella* strains were centrifuged, washed once in PBS and resuspended in twice the volume of GMM pH 4.5. 150 μl of the dilutions were transferred to 1350 μl of the corresponding medium and incubated at 37°C with shaking (160 rpm). To assess bacterial viability at different time points, serial dilutions were plated onto Tryptic Soy agar and colony forming units (CFU) were determined. Experiments were done at least three times in triplicates. The low-pH assay for RNA-Seq analysis was performed as follows: Pre-cultures of *B. microti* CCM 4915 and *B. suis* 1330 were grown overnight to stationary phase and diluted each in 40 ml TSB to reach an OD of 0.8 after 15 h of culture. Cultures were dispatched to 4 tubes with 10 ml each, centrifuged, and the pellets resuspended in 20 ml of GMM pH 4.5 or GMM pH 7.0 for the control conditions (2 tubes per strain and pH), pre-heated to 37°C. For each species and pH, the tubes were incubated at 37°C for 20 or 120 min. To preserve specific expression profiles, cultures were inactivated by the addition of 1/10 volume of a 30% phenol/ethanol solution and vigorous mixing, followed by centrifugation and storage of the bacterial pellets at −80°C.

### RNA Isolation and RNA-Seq

The total RNA of *Brucella* was isolated using the mirVana RNA Isolation Kit (Ambion), according to the manufacturer’s instructions. Each sample was treated with RNase-free DNase (Ambion) and tested by Polymerase Chain Reaction (PCR) for possible residual DNA contamination. When necessary, samples were treated again with DNase, prior to Agilent Bioanalyzer 2000 quality analysis of the RNA samples. rRNA depletion using the RiboZero Kit, generation of the cDNA libraries, and deep-sequencing was performed by Eurofins Genomics (formerly GATC, Germany) using an Illumina™ Hi-Seq 2500 platform and in-house protocols. The 51-bp reads were single-end, with a total yield of 34-64 million reads for the four *B. suis* samples and 38-62 million reads for the four *B. microti* samples.

### RNA-Seq Analysis

The CRAC software (Philippe et al., 2013) was used to strand-specifically map the reads to the reference genomes of *B. suis* 1330 (NC_004310.3 and NC_004311.2 for chromosomes I and II, respectively) and *B. microti* CCM 4915 (NC_013119.1 and NC_013118.1 for chromosomes I and II, respectively), and to filter out multi-aligned reads. 96-98% of the total mapped reads of all samples were single-aligned reads to the corresponding reference genomes, except for *B. suis* at pH 7/20 min (61%), and only these reads were kept for further analysis. Following mapping, the read counts for each gene were determined with the “featureCounts” software (Liao et al., 2014). Based on the CRAC output, normalization and differential gene expression analysis for the two different pH-conditions in each species were performed with DESeq (Anders and Huber, 2010), calculating the ratios of normalized reads at pH 4.5/normalized reads at pH 7.0, and the corresponding log2-values of the fold-changes, for each species and time point (20 min or 2 h). A threshold of ≥1.5 or ≤−1.5 was fixed for the log2-fold change. The sequencing reads from this study were deposited in the SRA database (NCBI) under the accession number PRJNA644280.

The genes selected on the base of the ≥ 1.5/≤ −1.5 log2-fold change threshold were classified by assigning the predicted proteins to their respective Cluster of Orthologous Groups (COG), using the Genoscope MicroScope platform from the French Sequencing Center^1^ (Vallenet et al., 2020), and according to *B. suis* 1330 and *B. microti* CCM 4915 operons predicted in the Database of prokaryotic OpeRons (DOOR) (Mao et al., 2009). In addition, a search for genes reported as virulence-associated was performed using the Pathogen-Host Interaction Data Integration and Analysis System (PHIDIAS) – Virulence Factors (Victors)^2^ (Sayers et al., 2019), as well as on the Virulence Factor DataBase (VFDB)^3^ (Liu et al., 2019). The mapping tool available at the Pathosystems Resource Integration Center (PATRIC)^4^ allowed the screening for possible clusters in the expression profiles. The metabolic pathway assessment was performed using the Kyoto Encyclopedia of Genes and Genomes (KEGG) Pathway Database.^5^

### Quantitative Reverse Transcriptase Polymerase Chain Reaction

To validate RNA-seq results, 95 differentially expressed candidate genes were selected and their expression changes at the pH-values and time points chosen were confirmed by Quantitative Reverse Transcriptase PCR (RT-qPCR). Primers were designed with the Primer3 software (Supplementary Table 1). Complementary DNA (cDNA) was obtained by reverse transcription of 1μg of total RNA in a final reaction volume of 20 μl, containing 4 μl SuperScript VILO Master Mix, at 42°C for 90 min. The working sample of cDNA was diluted 1:20 (2.5 ng/μl). RT-qPCRs were performed in triplicate per sample and experimental condition, using a Light Cycler™ 480 qPCR machine (Roche) and SYBR Green I Master (Roche) in a final volume of 1.5 μl per reaction. The reference gene for 16S-rRNA (*rrs*) was amplified in parallel for normalization. 396-wells microplates were prepared with the assistance of an Echo 525 Liquid Handler (Labcyte Inc.) at the Montpellier GenomiX (MGX) Platform. For each gene tested, the mean calculated threshold cycles (Ct) were averaged and normalized to the Ct of the 16S-rRNA gene used for reference. Calculation of the fold change using the ΔΔCt method was based on the normalized Ct (Hanna et al., 2011).

### Construction and Complementation of *Brucella* Mutant Strains

Mutant strains of *B. microti* were constructed by target gene deletion and replacement with a kanamycin resistance cassette obtained from pUC4K (Amersham Biosciences). Briefly, a fragment containing 2 homology regions at the 5’- and 3’-ends of the gene of interest was generated by overlap extension PCR (Heckman and Pease, 2007). This fragment was cloned into pGEM-T Easy (Promega; non-replicative in *Brucella*) and amplified in *E. coli* DH5α prior to insertion of the kanamycin resistance cassette into the unique restriction site *Stu*I and introduction into *Brucella* by electroporation, as described previously (Köhler et al., 1996). To select for allelic exchange mutants, Kan*^R^* colonies were checked for sensitivity to ampicillin. Allelic exchange in Kan*^R^*/Amp*^S^* clones was validated by PCR. Homologous complementation of *B. microti* mutants was achieved by transformation with the replicative *E. coli*-*Brucella* shuttle vector pBBR1-MCS (Kovach et al., 1994) carrying the intact sequences of the genes of interest. For *B. suis* 1330, pBBR1-AMP, containing a *bla* gene conferring resistance to ampicillin and replacing the chloramphenicol resistance marker of pBBR1-MCS by insertion into the *Nco*I-*Aat*II restriction sites, was used. DNA-fragments were obtained by PCR amplification using *Pfx* high fidelity DNA polymerase (Life Technologies) with primers containing restriction sites *Kpn*I/*Sac*I, followed by insertion into pBBR1-MCS. Primers used for gene deletion and complementation of *Brucella* strains are listed in Supplementary Table 2.

### Infection of J774-Murine Macrophage-Like Cells

Experiments were performed in triplicate as described previously, using J774A.1 murine macrophage-like cells at a multiplicity of infection of 20 bacteria per cell (Burkhardt et al., 2005). At defined time points, cells were lysed in 0.2% Triton X-100, and viable intracellular bacteria were determined after plating serial dilutions of lysates on TS agar and incubation for 3 days at 37°C.

### Atomic Force Microscopy

Tryptic Soy broth (TSB) cultures of *B. microti* were treated following the protocol described for acid stress assays and incubation in GMM for 6 h at pH 4.5 or pH 7.0. 1.5 ml of cultures were centrifuged, and the pellets washed with 0.22 μm-filtered PBS, prior to resuspension in 1 ml of 2.5% glutaraldehyde and incubation for 1 h. Fixed bacteria were washed with filtered PBS and resuspended in 150 μl. For Atomic Force Microscopy (AFM), FluoroDish™ cell culture dishes (World Precision Instruments, United Kingdom) were coated overnight at 4°C with 0.1% poly-L-lysine, washed with PBS, air dried and stored at 4°C. Bacteria were diluted 20-fold in filtered PBS and added to the functionalized dish. Topographic imaging in PBS at 20°C was performed on a NanoWizard IV AFM (JPK-Bruker) using a force-curve-based imaging mode (QI mode), with qp-BioAC cantilevers (Nanosensors, mean cantilever spring constant k_cant_ = 0.09 N/m). The applied force was kept at 300 pN, and a constant approach/retract speed of 80 μm/s (z range of 800 nm). Images were flattened with a polynomial/histogram line-fit with the AFM software.

### Statistical Analysis

Data from stress assays and infections were analyzed with the Student’s *t*-test, using Graph Pad Prism and Sigma Plot software. Data were expressed as means of at least three independent experiments with standard deviations. Differences were considered statistically significant when P-values were < 0.05.

## Results and Discussion

### Increased Resistance of *Brucella microti* to Intermediate Acid Stress at pH 4.5 *in vitro*

**FIGURE 1:**
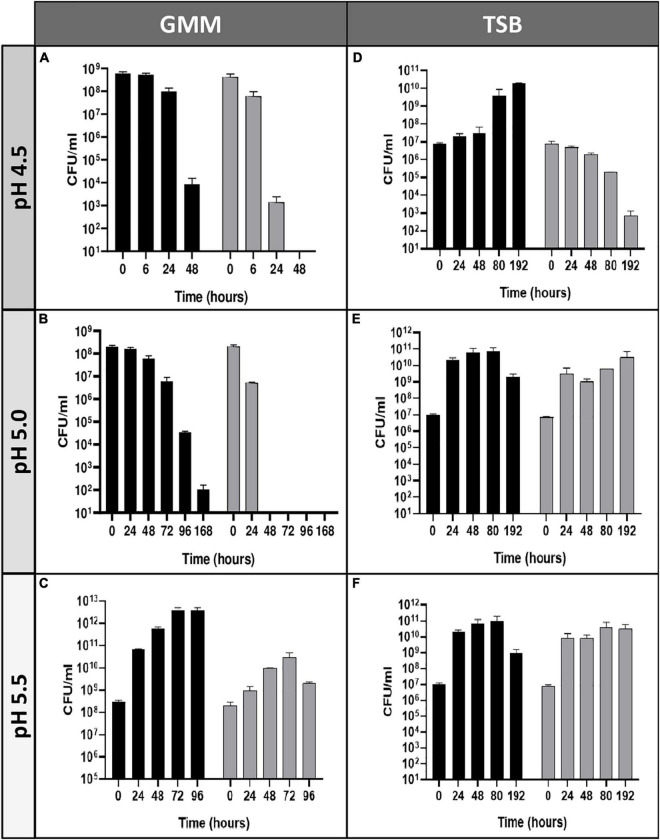
Survival and replication of *B. microti* and *B. suis* in minimal or complex medium at pH 4.5–5.5. Viability of *B. microti* (black bars) and *B. suis* (gray bars) in modified Gerhardt’s Minimal Medium **(A–C)** or Tryptic Soy broth **(D–F)** adjusted to pH 4.5, 5.0, and 5.5 was determined at different time points. Values are shown as means of 3 experiments ± standard deviations (SD).

Our group previously described that *B. microti* was more resistant to pH 4.5 in minimal medium than *B. suis* (Jiménez De Bagüés et al., 2010). To further explore this observation, comparative analyses of survival or growth rates of both species were performed in rich (TS) and minimal medium (GMM) at different pH values and time points (Figure 1). Whereas survival of *B. suis* and *B. microti* decreased in GMM at pH 4.5 and 5.0, with a significantly more rapid decline for *B. suis*, growth was observed for *B. microti* at the same pH values in complex TS broth. Growth of *B. suis* started at pH 5.0 in TS medium, and both species grew in GMM and TS broth at pH 5.5 (Figure 1). These results confirmed a better global adaptation of *B. microti* to intermediate acid stress at pH 4.0-5.0 than of the classical human pathogen *B. suis*, especially when coupled with a nutrient-poor environment.

### Transcriptome Analysis of Differential Gene Expression in *Brucella suis* 1330 and *Brucella microti* at pH 4.5 and pH 7.0 Reveals Common and Species-Specific Adaptation Profiles

Common and species-specific gene expression profiles of *B. suis* and *B. microti* during acid stress at pH 4.5 were obtained by RNA-Seq and analyzed following exposure of both species to pH 4.5 and pH 7.0 in GMM for 20 and 120 min, mimicking very early, possibly critical stages of adaptation to an acidified host cell vacuole during infection or to out-of-the-host environments such as fermented food products or acid soils. In contrast, previous transcriptome analyses of *Brucella* spp. at pH 4.4 generally focused on later time points, reflecting adaptation to an advanced stage of acidified endosomal BCV (Liu et al., 2016; Salmon-Divon et al., 2019).

**FIGURE 2:**
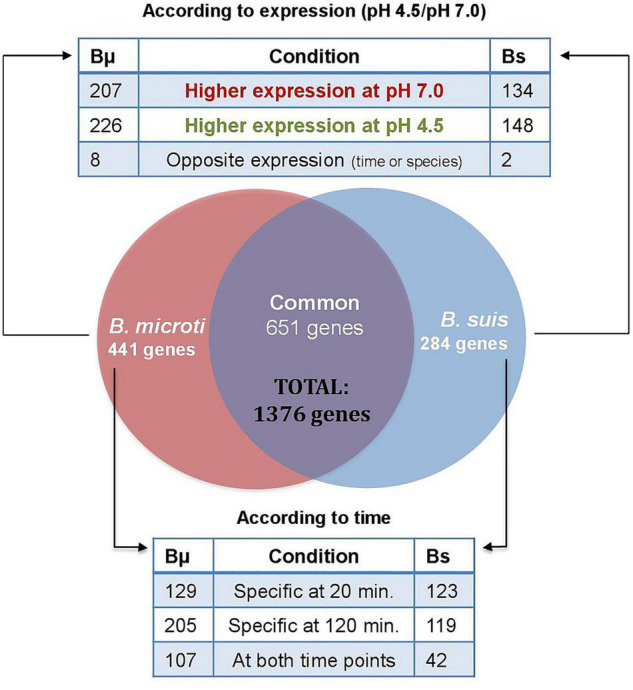
Distribution of differentially expressed genes in *B. microti* and *B. suis*. Numbers of genes differentially expressed in both species at pH 4.5/pH 7 were determined either according to the rates of expression at both pH-values (upper panel), or according to the time points where differential expression was significant (lower panel). Bμ: *B. microti*; Bs: *B. suis*.

Our results yielded a total of 935 and 1092 genes identified as being differentially regulated in *B. suis* and *B. microti*, respectively, at both time points (30–34% of the *Brucella* genome), applying as criterion a > 1.5 or <−1.5 log2-fold change to the ratio of the pH 4.5/pH 7.0 expression values. From these genes, 651 were commonly induced or repressed during acid stress, with 150 being significantly regulated in both species and at both time points, hereafter called “core genes” (Supplementary Table 3). Regarding the species-specific responses, only 284 genes (9% of the genome) were specifically regulated in *B. suis*, as opposed to 441 genes (14% of the genome) in *B. microti* (Supplementary Table 3). Whereas the distribution between conditions of the number of differentially expressed genes with higher rates of expression either at pH 4.5 or at pH 7 was similar in each species, the number of genes differentially regulated at 120 min and at both time points was clearly higher in *B. microti* (Figure 2).

Genomic distribution of the genes differentially expressed at pH 4.5 and pH 7.0 did not reveal any clustering and was homogeneous in both species (not shown).

### Quantitative Reverse Transcriptase Polymerase Chain Reaction Validates RNA-Seq Analysis

**FIGURE 3:**
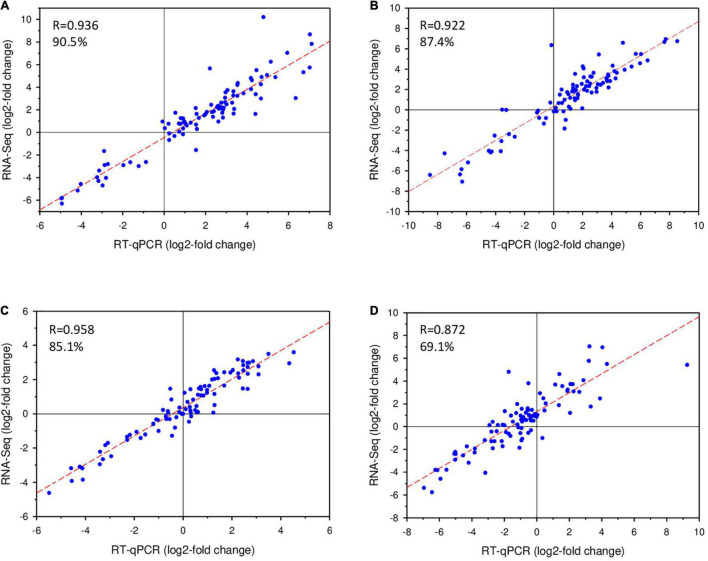
Validation of RNA-Seq data by RT-qPCR. Log2-values of fold-changes of gene expression at pH 4.5 and pH 7.0 detected by RNA-Seq were plotted against RT-qPCR data. Rates of validation are indicated as percentages in the graphs. **(A)** *B. microti*, 20 min; **(B)** *B. microti*, 120 min; **(C)** *B. suis*, 20 min; **(D)** *B. suis*, 120 min.

Reliability of the transcriptome analysis was assessed by RT-qPCR-based quantification of the RNAs from 95 representative genes. These genes were selected according to the following criteria: bacterial species; time points (20 or 120 min); COG groups; operon organization; annotated functions; log2-fold change ratios (significantly higher expression at pH 4.5 or 7.0). Expression of the genes was assessed with technical triplicates, using the 16S rRNA-gene as reference (constant expression rates under all conditions). RT-qPCR results were compared to those obtained by RNA-Seq, and correlations for each of the 4 experimental conditions are shown in Figure 3. Gene expression profiles identified by RNA-Seq were considered as validated, when expression rates determined by both approaches matched for one of the three conditions included in the analysis: significantly higher or lower expression at pH 4.5 than at pH 7, or non-significant differences in expression. The validation rates for the four experimental conditions chosen ranged from 70-90%, confirming the soundness of the RNA-Seq data (Supplementary Table 4).

As RT-qPCR was carried out with 7% of the total number of genes identified by RNA-Seq, a much larger sampling than that performed in other RNA-Seq studies described in the literature, the obtained validation rates were considered as solid for analysis of the RNA-Seq results, confirming the robustness of the experimental design and of the quantification procedures applied, in accordance with literature reports (Fang and Cui, 2011).

### Deciphering of Global *Brucella suis* and *Brucella microti* Transcription Profiles Contributes to the Understanding of the Increased Survival of *Brucella microti* at pH 4.5 *in vitro*

#### Core Genes

**FIGURE 4:**
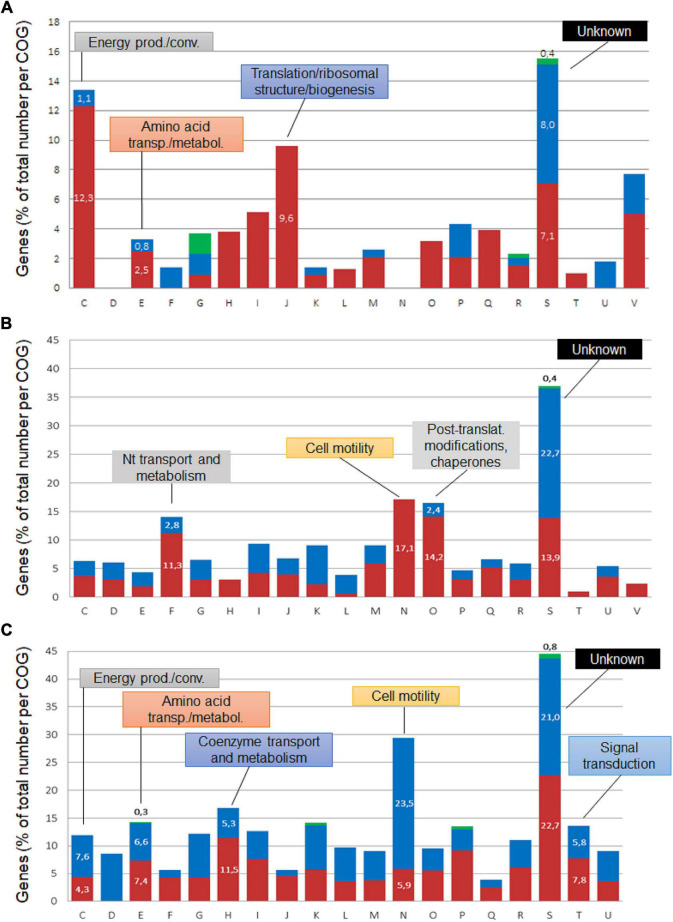
COG classification of *Brucella* genes regulated at pH 4.5. The differentially expressed core genes **(A)** and those specific to *B. suis* **(B)** and *B. microti* **(C)** cumulated for both 20 and 120 min were classified into Clusters of Orthologous Groups, and their percentages were determined with respect to the total number of genes affiliated to each group. Blue sections of the bars represent genes with higher expression at pH 7, red sections genes with higher expression at pH 4.5, and green sections correspond to genes with opposite expression over time. COGs: C: Energy production/conversion; D: Cell cycle control, cell division; E: amino acid transport/metabolism; F: Nucleotide transport/metabolism; G: Carbohydrate transport/metabolism; H: Coenzyme transport/metabolism; I: Lipid transport/metabolism; J: Translation/ribosomal structure/biogenesis; K: Transcription; L: Replication, recombination/repair; M: Cell wall/membrane/envelope biogenesis; N: Cell motility; O: Posttranslational modification/protein turnover/chaperones; P: Inorganic ion transport/metabolism; Q: Secondary metabolites synthesis/transport/catabolism; R: General function prediction; S: Function unknown; T: Signal transduction; U: Intracellular trafficking, secretion/vesicular transport; V: Defense mechanisms.

Of the 150 significantly regulated “core genes,” 108 genes were more expressed at pH 4.5, 38 at pH 7, and four in an opposite manner in both species (Figure 4A). The first group comprised genes encoding the following factors involved in energy production: F_1_F_0_-ATP synthase, cytochrome oxidases and NADH-quinone oxidoreductase (Supplementary Table 3). The activation of the F_1_F_0_-ATPase/ATP-synthase has been reported under acid conditions in bacterial species such as *E. coli* (Sun et al., 2012) and *Listeria monocytogenes* (Cotter et al., 2000). In our study, the induction of three of the F_1_F_0_-ATPase/ATP-synthase genes was observed: *atpG*, *atpD*, and *atpC*, the latter encoding the subunit participating in proton translocation. *atpC* activation in *Brucella* fits with previous reports in other bacteria, where its activity is presumed to act directly as a mechanism for intracellular proton extrusion, consuming ATP (Lund et al., 2014). However, due to the dual function of this machinery acting also as an ATP-synthase, it can generate ATP using an inward-directed proton-motive force, suggesting that the enzyme may also play a role in the production of the energy required for the activities of ATP-dependent proteases, chaperones or transporters involved in diverse acid resistance and repair mechanisms (Lund et al., 2014). In *E. coli*, the ATP production capacity was reported to be essential for acid resistance (Sun et al., 2011). Likewise, increase in intracellular ATP concentration during rapid environmental shifts such as a switch from neutral pH to pH 3.5, has been reported in *E. coli*, *Pseudomonas putida* and *Bacillus subtilis* (Albert and Brown, 2015). The potential importance of an alternating ATPase/ATP-synthase activity is evidenced by its activation under acid and alkaline stress in *E. coli*, demonstrating its versatile role in bacterial pH homeostasis (Maurer et al., 2005). However, the mechanisms remain to be elucidated, and deepening the understanding of the ATPase/ATP-synthase role in *Brucella* may contribute to a better comprehension of acid resistance and physiology.

The induction of cytochrome oxidase genes, mainly *cco* encoding the cytochrome *cbb*_3_ oxidase, was in line with acid stress resistance, as cytochrome oxidases catalyze the reduction of O_2_ to water, acting thereby as a proton-pump (Pitcher and Watmough, 2004). In addition to the also acid-dependent expression of the NADH-ubiquinone oxidoreductase complex I (*nuoEFHIKLMN*), the major respiratory chain complex, the *cbb*_3_ oxidase may act as a complementary system in the export of protons. In agreement with these findings, genes encoding the Ccm complex (*ccmDEFGHI*) involved in cytochrome *c* biogenesis, and the FnrN transcriptional regulator (BMI_I653/BR0654) were also induced. FnrN is known to regulate the cytochrome *cbb*_3_ oxidase through an oxygen sensing mechanism (Loisel-Meyer et al., 2005), and expression was increased in both species at pH 4.5 after 20 min Moreover, the cytochrome *cbb*_3_ oxidase as well as FnrN have been linked to bacterial persistence in the host, as inactivation of their genes in *B. suis* results in strong attenuation in murine infection during the chronic phase (Jiménez De Bagüés et al., 2007). A relationship between the aerobic/anaerobic metabolism of *Brucella* and its within-the-host life is known, with interplays between different cytochromes (Loisel-Meyer et al., 2005; Jiménez De Bagüés et al., 2007; Abdou et al., 2013). According to our results, the acid pH signal may also play a role in these crosstalks, possibly acting as a trigger for certain respiratory and metabolic changes.

While an active ATP-synthase generates translocation of protons from the periplasm to the cytosol, these proton-pumping mechanisms contribute to pH homeostasis under acid stress conditions, exporting protons to the external medium. Additionally, the cytochrome *cbb*_3_ oxidase is tightly linked to the initiation of denitrification processes, which are activated during microaerophilic and anoxic conditions and allow the bacteria to use nitrogen oxides as electron acceptors under these conditions. Interestingly, the cytochrome *bd* ubiquinol oxidase genes *cydDAB*, related to the denitrification process in *B. suis* since their lack impairs nitrite utilization (Jiménez De Bagüés et al., 2007), were also more expressed at pH 4.5.

It must be taken into consideration that the acidic environment created in the experimental model used in this study leads to a potential drop of the dissolved oxygen concentration, due to the reaction of hydrogen ions with oxygen. This phenomenon most likely explains the activation of systems associated to microaerophilic conditions, such as cytochrome *cbb*_3_ oxidase and denitrification, during exposure to acid stress.

The increased expression of two genes encoding outer membrane proteins (Omp) was also part of the common response in GMM at pH 4.5: the gene coding for an OmpW family small outer membrane protein whose function remains unknown but is predicted to be a porin, and *omp31*, encoding one of the major Omp in *Brucella* more expressed at pH 4.5 under three of the four conditions (Cloeckaert et al., 2002) (Supplementary Table 3). The OmpW family proteins form eight-stranded beta-barrels with a hydrophobic channel, possibly for small-molecule transport across the membrane. They have been related to resistance to environmental stress and to phagocytosis in *E. coli* (Wu et al., 2013). Omp31 has also been linked to resistance to diverse stress types (e.g., peroxide, polymyxin) and it participates in intracellular survival (Verdiguel-Fernandez et al., 2017). As a transmembrane protein, it is in direct contact with the extracellular acidic environment, which may lead to rapid protonation and possible denaturation, followed by loss of function of Omp31. Considering the importance of these proteins in stress resistance, the increased expression of their genes at pH 4.5 might reflect a compensatory mechanism to renew the acid-denatured molecules, thus maintaining their outer membrane functions; these may involve charge balancing, osmoregulation and transport. Genes of other Omps such as OmpF and OmpC are also induced and play an important role in survival under acid stress (Sato et al., 2000). The closed state of OmpC is induced and stabilized when the cytoplasmic pH drops and acid stimulation reaches the Omps from the periplasmic space (Liu and Delcour, 1998). Moreover, polyamines such as cadaverine and putrescine, released under acid stress by the action of lysine and ornithine decarboxylases, respectively, also contribute to closure of the outer membrane porins, reducing general permeability of the membrane (Samartzidou et al., 2003). Hence, maintenance of the porins’ closed state can contribute to protection against internalization of hydrogen ions.

Interestingly, genes of both major pathways of histidine metabolism were found to be more expressed in both species during acid stress: the histidine biosynthesis pathway, encoded by the *his* operon with *hisA*, *hisF*, and *hisI* (core gene), and the histidine degradation pathway, encoded by the *hut* operon with *hutI*, *H*, *G*, *J*. Based on the log2-fold change ratios, the expression rates of the *hut* operon were higher in *B. suis*, reaching values >4.0. Each pathway has different biological implications and both are tightly regulated. Biosynthesis of histidine provides carbon, nitrogen and energy sources, whereas acid resistance mechanisms are linked to degradation of histidine, generating ammonia and glutamate, the latter also playing a role in osmotic regulation. The *hut* system is regulated by diverse stimuli such as histidine or urocanate availability and nitrogen-limiting conditions, and controlled by the Ntr system. Despite nitrogen abundance, *ntrB* and *ntrC* showed increased expression at low pH in both species, which may be explained by the potential role of Ntr in ammonia supply for other pathways, such as glutamate synthesis and polyamine metabolism. The increased expression of the histidine utilization pathway genes therefore reflected a potential adaptation to low-nutrient conditions, as well as an attempt to provide molecules participating in the regulation of the internal pH. The increased expression of genes involved in the L-histidine biosynthetic pathway may contribute to purine biosynthesis, since both pathways share common steps. This is in agreement with increased expression of purine biosynthesis genes under acid stress, observed specifically in *B. suis* and discussed later. Due to its capacity to function as a major proton donor or acceptor, histidine plays a central role at crossroads of diverse cellular functions in bacteria, from purine and nucleotide synthesis to signal transduction, and histidine decarboxylation has been described in acid adaption (Trip et al., 2012).

Another highlight among the group of core genes with an increased rate of expression at pH 4.5 in both species were the genes encoding urease, a well-known actor in acid resistance, where hydrolization of urea by urease yields CO_2_ and ammonia, which counteracts the acid pH by protonation (Sangari et al., 2007; Lund et al., 2014). However, the genes for the accessory proteins participating in the maturation process of the urease (*ureE*, *ureF*, and *ureG*) were expressed differentially only in *B. suis*, suggesting an increased activity in this species (Supplementary Table 3). In the absence of urea in the medium, ammonium sulfate as well as glutamate, glutamine, arginine and their derivatives may feed the system and are possible NH^4+^-sources for the urea cycle. In line with this strategy is the activation of the *hut* operon, which encodes factors involved in degradation of histidine to yield ammonia and glutamate (Bender, 2012). The activation of the *hut* operon may also explain the lack of induction of the T4SS at these early time points of acid stress: *hut* operon repressor HutC is a positive regulator of the expression of the *virB* operon (Sieira et al., 2010). Hence, as long as the *hut* operon remains active, expression of the *virB* operon cannot be supported by the action of HutC.

A total of 38 core genes showed higher expression at pH 7.0 (Supplementary Table 3). Some of these genes encode key enzymes involved in gluconeogenesis: phosphoenolpyruvate carboxykinase (*pckA*), fructose-1,6-bisphosphatase (*fbp*), and fructose-1,6-bisphosphate aldolase (*fbaA*). A possible explanation for this metabolic down-regulation at pH 4.5 could be the favoring of other metabolic pathways such as the pentose phosphate pathway, the major pathway of hexose and pentose catabolism in most *Brucella* species. Moreover, in *B. microti* the genes encoding fructose 1,6-biphosphatase II, (*glpX*) participating in gluconeogenesis, and malic enzyme, catalyzing the conversion of malic acid to pyruvate with production of NADPH and linking glycolysis and gluconeogenesis with the tricarboxylic acid cycle, were also less expressed (Supplementary Table 3; *B. microti*-specific genes). The lower expression of these genes at pH 4.5 further reduced the gluconeogenic pathway during acid stress. Interestingly, the gene encoding malic enzyme is annotated as pseudogene in *B. suis*, resulting in modifications of the so-called phosphoenolpyruvate (PEP)-pyruvate-oxaloacetate node that regulates the major carbon metabolic pathways, but also in the possible loss of a pathway allowing generation of the key metabolic cofactor NADPH. In addition, of the four core genes showing opposite regulation in the two species, three encode sugar transporters.

#### *Brucella microti*- and *Brucella suis*-Specific Acid-Responsive Genes

In addition to the acid-responsive core genes, each species possessed a specific set of genes whose expression was modified by the acid stress. The genes specific to each species at 20 and 120 min were classified into Clusters of Orthologous Groups (COG) to predict possible functions, and their percentages were determined with respect to the total number of genes affiliated to each group (Figures 4B,C). Group S was the most abundant, but several groups with known functions were also identified as harboring an important number of acid stress-dependent factors (Figures 4B,C): C (energy production and conversion); E (amino acid transport and metabolism), F (nucleotide transport and metabolism); H (coenzyme transport and metabolism); N (cell motility); O (post-translational modification, protein turnover and chaperones).

#### *Brucella microti*-Specific Acid-Responsive Genes

The analysis of the *B. microti*-specific acid stress response gave some important clues on the adaptation of the pathogen to these harsh environmental conditions (Supplementary Table 3). A remarkable feature was the increased expression at pH 4.5 of the genes encoding RNA polymerase sigma factors sigma 24 (*rpoE*) and sigma 32 (*rpoH2*), in accordance with previous reports where *rpoE* activates *rpoH2* (Roncarati and Scarlato, 2017). This strengthened the hypothesis of a species-specific processing of the acid stress signals in *Brucella*, with a possibly earlier response in *B. microti*. The *B. microti*-specific increased expression of a two-component response regulator-encoding gene (BMI_I1679), annotated in some *Brucella* species as PhyR, was in line with the increased expression of *rpoE*: Both are central elements of the general stress response in alpha-proteobacteria and have been described as required in *B. abortus* for *in vitro* stress survival and chronic murine infection (Kim et al., 2013). Moreover, *rpoE* has been reported to be expressed consecutively to envelope damage detected by sensing of misfolded Omps, and both factors are involved in maintaining the integrity of periplasmic and outer membrane components (Raivio and Silhavy, 2001). In *B. melitensis*, RpoE1 also acts as repressor of the flagellar genes (Ferooz et al., 2011). Hence, increased expression of the corresponding gene at pH 4.5 exclusively in *B. microti* may explain the lack of activation of flagellar genes in the atypical species during acid stress, whereas flagellar genes are more expressed in *B. suis* (see below).

**FIGURE 5:**
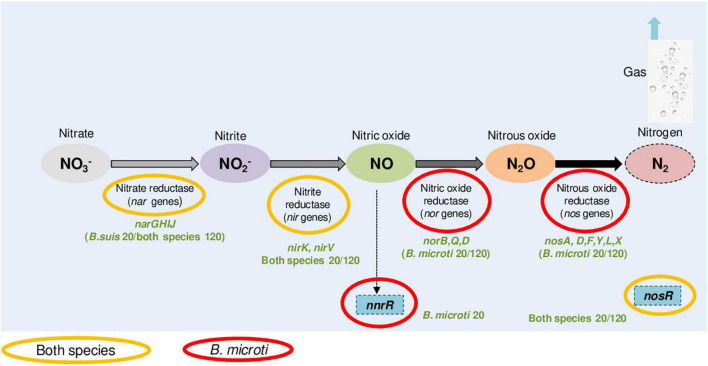
*B. microti* and *B. suis* genes of the denitrification pathway with increased expression at pH 4.5. Color codes indicate the species and the experimental time points (20 or 120 min) for each of the genes identified as being more expressed at pH 4.5 than at pH 7.

Another particular feature of the specific *B. microti* response to acid stress was the strong activation of nitrogen metabolism genes, especially the denitrification pathways (Supplementary Table 5), with 19 members one of the strongest sets of positively regulated genes at pH 4.5. This observation was in agreement with the increased expression at pH 4.5 of the *cbb*_3_ cytochrome oxidase components, linked to denitrification and induced by low oxygen concentrations and by exposure to reactive nitrogen species and nitrite. Despite the fact that the genes encoding nitrate reductase Nar and nitrite reductase Nir, catalyzing the first two steps of denitrification, were more expressed at pH 4.5 in both species, acid-induced expression of *nor* and *nos*, encoding nitric oxide and nitrous oxide reductases, was specific to *B. microti* (Figure 5). As a consequence, *B. microti* avoided the possible accumulation of harmful NO, which may, in contrast, affect *B. suis*. This evidence suggested a differential regulation of the denitrification pathways under acid stress, and hence a species-specific control of the anaerobic/microaerobic respiration. Specific activation of *nor* and *nos* operons is likely to be based on the pH-mediated decrease of dissolved oxygen in acid medium, suggesting that *B. microti* may have a faster adaptive response under acid-induced low oxygen conditions. Interestingly, the *Brucella nar* operon is also induced during the stringent response, constituting another overlapping factor in multiple stress responses (Hanna et al., 2013), and the denitrification pathway has been related to *Brucella* virulence in mice and intracellular resistance to NO (Haine et al., 2006; Loisel-Meyer et al., 2006).

Furthermore, acid-dependent regulation of genes involved in methionine metabolism was significantly different between both pathogens: The acid-induction of four genes (*ahcY*, *metH*, *metF* and *sahR*) involved in the S-adenosylmethionine (SAM)-S-adenosylhomocysteine (SAH) cycle suggested a higher methylation activity in *B. microti*, since SAM, a nucleoside generated from methionine, is a coenzyme for methyltransferases (Supplementary Table 6). It is also required for the synthesis of polyamines, quorum sensing molecules and production of vitamins, demonstrating the important role of SAM in bacterial metabolism. This enhanced activity of the SahR regulon may also be related to the higher expression of the genes *bioA* and *bioB*, coding for biotin-ligase and dethiobiotin synthase, respectively, and to the induction of the cobalamin (B12) synthesis pathway (precorrin-2 methyltransferase, *cbiG*, *cbiQ*, *cobN* and cobalamin synthesis proteins) in *B. microti* (Supplementary Table 3). Increased DNA-methylation activity *via* SAM impacts regulation of gene expression, conservation of genome integrity and cell cycle regulation, and SAM has also been associated to riboswitch-dependent genetic regulation (Batey, 2011). An active methionine metabolism in *B. microti* may be related to a higher rate of transcriptional/translational activity, but also points out differences to *B. suis* concerning regulation of the sulfur metabolism, which most likely participates in redox homeostasis through biosynthesis of thiol antioxidants such as glutathione, cysteine, and homocysteine, also required during acid stress.

Overall, 4–7% of the putative protein-encoding genes in *Brucella* genomes are pseudogenes. Pseudogenization can lead to many phenotypical differences and play an important role in *Brucella* host specificity and virulence (Chain et al., 2005). Interestingly, expression of 15 genes reported as being pseudogenes exclusively in *B. suis* was influenced by acid stress in *B. microti* (Supplementary Table 7): genes encoding choline dehydrogenase (BMI_I1654), cold-shock protein CspA and D-lactate dehydrogenase Dld were of main interest. The choline dehydrogenase is an enzyme linked to osmoprotection, catalyzing the glycine betaine synthesis from choline oxidation. Glycine betaine has been reported to protect also from heat shock, acting as a protein stabilizer and contributing to protein renaturation (Caldas et al., 1999). Other osmoregulation mechanisms are also coordinately activated in *B. microti*, such as glycine betaine/L-proline ABC transporters, while the gene encoding the mechanosensitive channel MscL, undergoing conformational change under membrane stretching, is repressed in *B. microti*, possibly due to ion permeability when the channel is open (Supplementary Table 3). Due to the intricate regulation of osmotic and acid stress responses, characterized by the overlapping functions of molecules involved in both responses such as K^+^, glutamate and other osmolytes, pH-regulating mechanisms can have an impact on osmotic pressure through membrane permeability changes, denaturation of proteins and generation of metabolites such as ammonia, urea, GABA, etc. These variations, possibly resulting in osmotic imbalance, may activate the adequate systems to cope with this additional stress.

The identification of certain pseudogenes in *B. suis* may also give some clues about metabolic and physiologic differences between both *Brucella* species, looking at the examples of the genes encoding thioredoxin reductase and D-lactate dehydrogenase Dld (Supplementary Table 7). Thioredoxin reductase, whose expression was downregulated at pH 4.5 in *B. microti*, is involved in ribonucleotide reduction. Hence, its lack in *B. suis* may imply the use of alternative pathways to produce deoxyribonucleotides. The D-lactate dehydrogenase (Dld), activated by *B. microti* early at pH 4.5 but downregulated later, may play a role in electron transfer during anaerobic conditions. The loss of the D-lactate dehydrogenase expression in *B. suis* in addition to less efficient denitrification suggests a better adaptation of *B. microti* to anaerobic or microaerophilic conditions. Interestingly, in opposition to the situation observed in *B. suis* with 15 pseudogenes which were acid-stress regulated in *B. microti*, none of the acid stress-regulated genes found in *B. suis* were pseudogenes in *B. microti*, despite the fact that a total of 79 pseudogenes are present in *B. microti* versus 109 pseudogenes in *B. suis* (NCBI Genome database; Audic et al., 2009). It is conceivable that the loss of certain genes during host-adaptation impairs *B. suis* acid stress resistance, when compared to the more ancestral and free-living species *B. microti*. These results therefore give additional evidence that gene mutations shape the physiology of *Brucella* species.

In our study, genes encoding the type IV secretion system components were not characterized by increased rates of expression under acid conditions (Supplementary Table 3), which was unexpected, since acid stimulation and nutrient-poor medium are known to trigger *virB* expression (Boschiroli et al., 2002). *virB1* and *virB6* were significantly less expressed at pH 4.5 in *B. microti* at both time points, while *virB7* was less expressed under acid conditions in *B. suis* at 20 min The low ratios of transcription at pH 4.5 *versus* pH 7.0 may be explained by the fact that the *virB* operon is induced at pH 4.5 at later time points only.

Interestingly, three genes corresponding to cold shock proteins (*csp*), including *cspA*, showed higher expression at pH 4.5 in *B. microti*, two of them specifically. In line with the described cold shock responses (Zhang et al., 2018), two helicases and a VacB/RNAse II exoribonuclease, possibly the RNAse R described to act with CspA, also presented increased expression (Supplementary Table 8). In addition, the *B. melitensis* homolog of the *csp* gene BMI_I1528 was reported to play an important role in *Brucella* virulence and stress adaptation (Wang et al., 2014).

The sulfur mobilization (*suf*) operon, considered as essential in *Brucella* (Sternon et al., 2018), was also more expressed in *B. microti* at pH 4.5. It is required for the assembly of Fe-S clusters, acting as cofactors in diverse metabolic reactions, as electron carriers in redox reactions, and in gene regulation and DNA repair and replication. It is activated during iron limitation or oxidative stress (Blahut et al., 2020). Due to protonation and denaturation induced by acid stress, Fe-S cluster-containing proteins as well as DNA are likely to be damaged, explaining the increased expression under acid conditions of the *suf* operon in *B. microti* (Supplementary Table 9). Moreover, the ATPase function of SufC may also have a role as a proton pump in acid stress, suggesting different ways for the Fe-S clusters in the bacteria to cope with redox state disturbances. Under acid stress, expression profiles of *B. microti* and *B. suis* were different for iron and sulfur acquisition pathways, including higher expression in *B. microti* of genes coding for certain iron transporters, sulfur-containing amino acid metabolism and the ferritin-like protein Dps.

#### *Brucella suis*-Specific Acid-Responsive Genes

The identification of different groups of genes specifically regulated in *B. suis* sheds light not only on the mechanisms this pathogen has set up to respond to acid stress, but also on the physiological alterations the species may be exposed to. The higher rates of expression at pH 4.5 of genes participating in purine biosynthesis (*purE*, *purH*, *purC*, *purL*), peptidoglycan biosynthesis (*nagB*, *mraY*, *murE*, *murD*, *murF*) and lipid A biosynthesis (*lpxL*) suggested increased levels of structural damages of the DNA, cell wall and outer membrane, when comparing to the expression rates of the same sets of genes in *B. microti*, and could be a clue to the observed reduced survival of *B. suis in vitro* at pH 4.5 (Supplementary Table 3).

DNA damage, known to be one of the harmful effects of acid stress profoundly altering normal cell physiology, is mediated by DNA depurination, with rates increasing as the pH decreases (Raja et al., 1991). Increased expression of the purine biosynthesis operon *pur* may therefore contribute to generate nucleotides for the repair of damaged DNA. In line with this observation was the increased expression of genes at pH 4.5 in *B. suis* encoding the ribonucleotide-diphosphate reductase (RNR), involved in the synthesis of deoxyribonucleotides from ribonucleotides required for DNA synthesis during replication or repair processes (Torrents, 2014). Under our experimental conditions, *B. suis* increased the expression of the genes encoding the RNR subunits alpha (*nrdE*) and beta (*nrdF*) at pH 4.5, whereas *B. microti* showed a higher rate of expression of these genes at pH 7.0 (Supplementary Table 3). Since this system plays an essential role, its operon must be tightly regulated to ensure correct RNR concentrations and balanced dNTP levels (Torrents, 2014), a fact that adds to the biological relevance regarding different expression levels observed in both *Brucella* species. In the absence of growth of *Brucella* in minimal medium at pH 4.5, increased expression of *nrdE* and *nrdF* suggested an active DNA-repairing process in *B. suis*, as a response to increased sensitivity to acid stress at pH 4.5.

Genes involved in the peptidoglycan-recycling pathway and in peptidoglycan (PG) synthesis also showed increased expression specifically in *B. suis* at pH 4.5, among which *nagB*, encoding glucosamine-fructose-6-phosphate aminotransferase, and genes encoding the three main ATP-dependent amino acid ligases MurD, MurE, MurF and phospho-N-acetylmuramoyl-pentapeptide transferase (MraY) (Supplementary Table 3). The potential acid-related damage to PG in *B. suis* may affect bacterial physiology and hamper *Brucella* survival. PG plays a crucial role in maintaining bacterial shape and turgor pressure while allowing nutrient transport. In addition, the movement of the bacterial cytoskeleton-like elements has been shown to be tightly interdependent with PG synthesis, which is also regulated by lipoproteins anchored to the inner layer of the outer membrane, suggesting that PG synthesis is governed by internal and external signaling (Typas et al., 2011). These links may give clues about possible envelope rearrangements during acid stress, when the outer membrane is damaged by high H^+^-concentrations. The stronger expression of purine and peptidoglycan biosynthesis genes at acid pH in *B. suis* was consistent with studies performed in *E. coli* (Shayanfar et al., 2018), suggesting their participation in a general acid-stress response in certain Gram-negative bacteria. In the outer membrane, acid pH induces loss of stabilization of LPS as a consequence of increased phosphorylation of lipid A (Li et al., 2020). Therefore, the increased expression of *lpxL* encoding lauroyl acyltransferase and participating in lipid A biosynthesis may represent, alongside with PG and purine synthesis, an attempt to maintain cellular integrity during acid stress in *B. suis*.

Furthermore, a significant increase in expression of *entC* encoding isochorismate synthase was observed at pH 4.5 in *B. suis* (Supplementary Table 3). This enzyme participates in the 2,3-dihydroxybenzoic acid siderophore synthesis pathway, catalyzing the conversion of chorismate to isochorismate and it is responsive to environmental iron availability. The increased expression of a gene of the enterobactin operon suggested an important need for iron in *B. suis* during acid stress, which can be explained by the fact that iron is an essential cofactor for the assembly of Fe-S cluster proteins and ribonucleotide reductases.

The increased expression of the erythritol metabolism-related genes under acidic conditions only in *B. suis* is remarkable, since this sugar alcohol is a major substrate in brucellae. Erythritol feeds the pentose phosphate pathway and is considered as one of the determinants for the tissue tropism of *Brucella* toward the host’s reproductive tract (Barbier et al., 2014). In addition, genes *eryB* and *eryC* are necessary for intramacrophagic growth (Köhler et al., 2002). This acid pH-mediated differential expression of *ery* genes may reflect specific metabolic adaptation and mechanisms of regulation, possibly having an impact on the within-the-host life of *Brucella*.

#### Function-Based Expression Analysis, Independent of Species-Specificities

The identification of acid-dependent genes encoding factors interacting with the bacterial envelope added information on the strategies the pathogen has set up to create an efficient barrier against adverse effects of low pH. Some of these genes have already been presented above. To assess the possibility of an eventual acid stress-induced activation of repair processes in the outer membrane, a survey of acid stress-regulated genes potentially associated to such processes was carried out with the threshold-selected 1376 genes. Eleven of them encoded factors involved in LPS or outer membrane proteins biosynthesis (Supplementary Table 10). The modulation of outer membrane compound synthesis may have significant impacts on bacterial survival at acid pH and on infection biology. The LPS, major component of the outer membrane, is well-recognized to be crucial for host-pathogen interactions, and microorganisms adapt the LPS structure in response to the environmental conditions, including acid stress (Li et al., 2020). Regarding the outer membrane proteins and their function as permeable channels as well as their role in sensing, the increased expression of Omp31-1, Omp31-2 and the OmpW family protein may be an indicator of additional repair mechanisms in both species.

Remarkably, in *B. suis* the acid stress induced, at the early time point of analysis, an almost species-specific, significant increase in the expression of 14 genes belonging to the flagellar machinery (Supplementary Table 11). A heatmap of the expression profiles of the genes assigned to COG N for both *B. suis* and *B. microti* is shown in Supplementary Figure 1A, giving a comparative overview of differential expression of functionally related genes for all experimental conditions applied. Most genes encoding flagella components showed a significantly higher expression at pH 7.0 in *B. microti* at 120 min, whereas a transient trend for increased expression at pH 4.5 was observed for the same genes in *B. suis* at 20 min. *Brucella* species normally do not display a motile phenotype, with the exception of *B. melitensis* and bullfrog and Pac-Man frog isolates, where a flagellum was reported (Fretin et al., 2005; Soler-Llorens et al., 2016; Al Dahouk et al., 2017). The biological function of these genes in *Brucella* is still unknown, however, the bacterial flagellum contains a transmembrane Type III secretion system, including the ATPase encoded by *fliI*, evolutionarily related to the F_1_F_0_-ATPase. This suggested that this apparatus or part of it may function as an additional proton-pump under acid stress in *B. suis*.

In addition, 17 genes encoding proteins of the classical heat shock response showed higher expression at acid pH (Supplementary Table 12). Ten of these were specifically more expressed in *B. suis*, suggesting that the classical pathogen was activating more chaperone- and protease-dependent mechanisms of protein degradation or re-folding. Increased expression of the genes encoding the chaperone pairs DnaK/J and GroEL strongly suggested that acid stress-mediated protein misfolding or damaging occurred in a more significant way in *B. suis* than in *B. microti*. Genes encoding heat shock-induced proteases, important in degradation of aggregated or denatured proteins and in the removal of potentially toxic degradation products, also showed an increased expression under acid stress in *B. suis*. In bacteria, Lon is the major protease participating in degradation of misfolded proteins under stress conditions, but as a player in the protein quality-control system, it also degrades proteins under physiological conditions. Exemplified by ClpB, DnaK, and DnaJ, proteases can also assist chaperones unfolding and renaturing protein aggregates. Other genes more expressed at pH 4.5 encode the ATP-dependent protease Hsl forming a proteasome-like degradation complex (Supplementary Table 12). Presented as a heatmap, the divergence between *B. suis* and *B. microti* in acid-dependent regulation of these COG O-associated genes encoding factors involved in posttranslational modification, protein turnover and chaperones, was evident: At 120 min, 11 of the 47 differentially expressed genes were regulated in an opposite manner in the two pathogens, with a clear domination of higher expression at pH 4.5 in *B. suis*, whereas the majority of the genes more expressed at pH 7.0 were found in *B. microti* (Supplementary Figure 1B). General increase of expression of genes involved in protein turnover and in chaperone biosynthesis at low pH in *B. suis* gave rise to the speculation that under these conditions, this pathogen was more exposed to harmful protein denaturation than *B. microti*. Increased expression of certain heat shock factors (GroEL, DnaK) during acid stress has been previously reported for *Brucella* (Köhler et al., 1996; Teixeira-Gomes et al., 2000). Remarkably, expression of heat-shock response sigma factor-encoding *rpoH* and *rpoE* genes was not increased under acid conditions in *B. suis*, while *B. microti* showed an increase already during early exposure (see above). Our results showed that the acid stress-induced response in *B. suis* has the traits of a marked heat shock response, with several factors shared by the responses to different stress types: DnaK, related to heat and hyperosmotic stress; HslO, known to be redox-regulated and to participate, together with LbpA, in oxidative and heat stress; Lon, involved in the heat-shock response and in drug resistance processes. These findings are in agreement with a previous report on the induction of a heat shock-like response by *pmf* dissipation in *E. coli* (Gage and Neidhardt, 1993), fitting also with the hypothesis of increased envelope damage in *B. suis* at pH 4.5.

Furthermore, RNA-Seq results revealed that transport functions of *Brucella* were highly impacted by acid stress, and species-dependent specificities of transport activities may influence acid stress responses. A total of 180 genes encoding transporters had an altered expression profile under acidic conditions. Among those, a vast majority of 154 genes were found to be associated to ABC-transporters, with more than 50% of them affected, and 142 out of the 180 genes belonged to 71 predicted operons (Supplementary Table 13). 67 genes were significantly regulated in *B. microti* only. Interestingly, a general tendency of increased expression of genes encoding branched-chain amino acid transporters was observed in *B. microti* at pH 4.5 after 120 min of exposure. Similarly, 3 genes of a glycine betaine/L-proline transport system described for *Brucella*, as well as a sulfate ABC transporter and the permease component of a taurine transporter, were more expressed in *B. microti* under these conditions (Jenner et al., 2009). The activation of the glycine betaine/L-proline system reflected a potential need for these osmolytes, since pH homeostasis is interconnected with osmoregulation and the redox state of the cell: H^+^ extrusion has to be compensated to maintain osmotic and redox homeostasis. Higher expression of sulfate and taurine transporters suggested a more active sulfur metabolism in *B. microti* during acid stress. Taurine, an amino acid-like organosulfonate, is an osmolyte that also participates in osmoregulation. ABC transporters have been associated with diverse physiological processes from DNA-repair to gene regulation, and are related to virulence (Jenner et al., 2009). 10 of the identified ABC transporter-encoding genes are listed in the PHIDIAS Victors database as virulence-associated. Finally, the general induction of ATP hydrolysis-based transporters might contribute to bacterial pH homeostasis through proton expulsion by the ATPase components of these systems. In some cases, when the transported molecule is an osmolyte or compatible solute (sugars, polyols, amino acids), the process may result in a double win situation for the cell, expelling protons and importing a neutral molecule.

Eighty-one genes encoding transcriptional regulators and two-component systems (TCS) were identified as being differentially regulated in the two *Brucella* species (Supplementary Table 14). A lower expression of most transcriptional regulators was observed under acid stress with only 26 genes being more expressed at pH 4.5, of which 13 were specifically regulated in *B. microti*, 4 in *B. suis* and 9 in both species. The most represented regulator families were GntR and AsnC, followed by AraC, ArsR, MarR, and LysR. In addition, *betI* (BMI_I553/BR0554) was more expressed in *B. suis* but less expressed in *B. microti* after 120 min. BetI is a repressor of the glycine betaine synthesis pathway and of a choline transporter.^6^ This difference may reflect species-specific osmoregulation, with an increase of glycine betaine synthesis and transport in *B. microti* (see above). The genes with higher expression under acid conditions in *B. suis* encoded members of the DeoR, GntR and AsnC families. In *Brucella*, DeoR and GntR regulators are linked to host-pathogen interactions, but also involved in sugar metabolism and control of carbon and amino acid metabolism, or fatty and organic acids concentrations. In *B. microti*, genes coding for transcriptional regulators with increased expression under acid stress belonged to the ArsR, AsnC, IclR, LysR, OmpR and Ros/Muc families. The homologs of the *B. microti* Ros/MucR transcriptional regulator (BMI_I569) have been associated with virulence in *B. melitensis* and *B. abortus*, since their inactivation impairs intracellular growth and results in strong attenuation (Caswell et al., 2013). Despite the fact that these data must be interpreted with caution, because most of these regulators belong to large families with a wide array of possible functions in interconnected networks, such a difference between *B. suis* and *B. microti* in transcriptional regulation goes well with an earlier activation of virulence mechanisms in *B. microti*. Among the TCS, we identified two pairs of candidates with increased expression at pH 4.5 (Supplementary Table 14): BMI_II225/BMI_II226, the latter being a pseudogene in *B. suis*; BMI_I1535/BMI_I1537 at 120 min in *B. microti* only, encoding OmpR/EnvZ originally described as a porin regulator, controlling the outer membrane proteins OmpF and OmpC linked to acid stress response. In *Salmonella* and *E. coli*, OmpR/EnvZ participate in sensing of environmental signals such as osmotic and acid stress, and the intracellular acidification promoted by OmpR is necessary for T3SS expression (Chakraborty and Kenney, 2018). The genes BMI_II231 and BMI_II615, encoding potential TCS members with GGDEF-domains related to diguanylate cyclase- and phosphodiesterase-mediated biosynthesis and degradation, respectively, of the bacterial second messenger c-di-GMP, were acid pH-induced in *B. microti*, possibly indicating species-specific activation of the c-di-GMP second messenger functions linked to virulence, stringent response, motility, proteolysis, cell cycle and cell-cell communication (Römling et al., 2013). The complex functions of transcriptional regulators identified here necessitate specific transcriptomic studies to deepen knowledge on their biological roles under these environmental conditions.

#### Virulence-Related, Acid-Stress Responsive Genes

Several acid-responsive genes of *Brucella* have been previously reported as virulence-related, according to PHIDIAS Victors and VFBP databases. In our work, 69 virulence genes listed in these databases were identified as acid-stress responsive (Supplementary Table 15), out of 159 virulence-associated genes reported for *B. suis*. From the 69 genes, 28 were regulated in both species, 19 and 22 specifically in *B. suis* and *B. microti*, respectively. Altogether, 25 of the virulence-related genes were down-regulated, while 39 were more expressed under acid conditions, and 5 showed up- or down-regulation, depending on time-points or species. Species-specifically regulated virulence genes with increased expression under acid conditions were more numerous in *B. suis* (14/19) than in *B. microti* (10/22), indicating specific gene expression signatures, possibly in the species’ interactions with the host cell. The most represented COGs were the groups E (amino acid transport/metabolism), C (energy production/conversion) and G (carbohydrate transport/metabolism). As an example of genes listed in both databases, *rfbD* encodes the LPS O-antigen export system permease. It was more expressed at pH 4.5 in *B. microti*, suggesting that *B. microti* may preserve structural integrity of the envelope by activation of specific repair mechanisms, while *B. suis* engages into a larger response involving chaperones, proteases and potential DNA-repairing mechanisms.

Recently, Salmon-Divon et al. reported 773 *B. melitensis* 16M genes differentially expressed (fold change ≥ 2) between normal and low-pH conditions in TS broth at pH 4.4 after 4 h of incubation. Despite the differences in experimental conditions, 25 of these genes were also identified in our analysis as being differentially expressed simultaneously for both *B. suis* and *B. microti* (fold change ≥ 2.8), including 15 that were upregulated and 10 that were downregulated under acid-pH conditions. Another 12 and 6 genes were identified as being specifically regulated in *B. suis* and *B. microti*, respectively (Supplementary Table 16). The common induction of genes of the respiratory chain points out the importance of this system comprising proton pumps which maintain physiological conditions in the cytosol of *Brucella* species. Other highly conserved mechanisms of acid protection were induction of histidine degradation *via hut* genes generating protective ammonia and glutamate, and increased expression of urease-associated genes.

### Selection of Acid Stress-Induced *Brucella* Genes and Evaluation of Their Participation in Acid Stress Resistance at pH 4.5 and in Intramacrophagic Survival

Based on their predicted biological functions, their acid stress-induced expression rates, and their potential relationship to virulence or acid resistance mechanisms, 16 genes were selected for inactivation by homologous recombination (Table 1). All mutants were constructed in *B. microti*, aiming at the identification of factors contributing to the increased acid resistance of this atypical species versus *B. suis*, and then tested for *in vitro* acid stress resistance in minimal medium at pH 4.5 and for intracellular growth in J774 murine macrophages. Fourteen of the mutants, affected in the genes BMI_I1066, BMI_II893, BMI_I1677, BMI_II226, BMI_II385, BMI_II460, BMI_II847, BMI_I1652, BMI_I1679, BMI_I733, BMI_I686, BMI_II580, BMI_II225, and BMI_II898, did not show any reduction in survival as compared to the wild-type strain in minimal medium at pH 4.5 over an incubation period of 24 h (not shown). In the J774 murine macrophage infection model, characterized by transient acidification to pH 4–4.5 during the first phase of *Brucella* infection (Porte et al., 1999), none of the 16 mutants showed attenuation, and intracellular survival and replication profiles were identical to that of the wild-type strain for all time points [data not shown; (Jiménez De Bagüés et al., 2010)]. In addition, mutants in genes BMI_I426 and BMI_I2170 were also studied in human monocyte-derived macrophages from peripheral blood mononuclear cells, and intracellular replication profiles were identical to those in J774 cells (not shown).

**TABLE 1:** Genes selected for mutation in *Brucella microti.*

Gene ID (*Bs*/*B*μ)	Function/Protein	Deletion (nt/total)
BR1061/BMI_I1066	Zn-containing alcohol dehydrogenase	766/984
BR0423/BMI_I426	Cold Shock Protein CspA (Pseudogene in Bs)	159/210
BRA0899/BMI_II893	Ornithine cyclodeaminase ArcB	890/1077
BR1655/BMI_I1677	Sensor histidine kinase	705/936
BRA0229/BMI_II226	Two-component response regulator (Pseudogene in Bs)	481/669
BRA0388/BMI_II385	Heme-thiolate monooxygenase	1006/1164
BRA0463/BMI_II460	HlyD family secretion protein	634/945
BRA0853/BMI_II847	Hypothetical protein (putative permease of DMT family)	246/420
BR1634/BMI_I1652	Hypothetical protein (T4SS effector VceA)	197/318
BR1657/BMI_I1679	Two-component response regulator	694/795
BR0735/BMI_I733	Hypothetical protein (TIR domain-containing protein; T4SS effector BtpB)	731/834
BR0691/BMI_I686	Hypothetical protein (T4SS effector BspB)	193/255
BRA0586/BMI_II580	Hypothetical protein (virulence protein VirJ)	1014/1161
BR2149/BMI_I2170	DNA starvation/stationary phase protection protein Dps	389/498
BRA0228/BMI_II225	Sensor histidine kinase	1132/1341
BRA0904/BMI_II898	RNA-binding S1 domain-containing protein/Transcriptional accessory protein	2238/2304

*Bs: B. suis. Bμ: B. microti. nt: nucleotides.*

### Cold Shock Protein A and DNA-Binding Protein From Starved Cells Contribute to *Brucella microti* Acid Resistance at pH 4.5

In contrast to the 14 mutants described above, inactivation of genes BMI_I426 and BMI_I2170 significantly affected *B. microti* mutant survival in the *in vitro* acid stress model at pH 4.5. BMI_I426 encodes the cold shock protein CspA, previously studied in *E. coli* (Jiang et al., 1997), and BMI_I2170 codes for the “DNA-binding protein from starved cells” (Dps), also called DNA starvation/stationary phase protection protein, a nucleoid-associated protein (NAP) that participates in the arrangement of the bacterial chromosome (Calhoun and Kwon, 2011). Both *cspA* and *dps* have been originally related to other types of stress and can now be associated to acid stress response in *B. microti*.

The CSP are multifunctional, highly conserved, RNA-/DNA-binding small proteins of 65–70 amino acids in size, whose expression is mainly induced by temperature decrease. The cold shock domain contains 2 nucleic acid-binding motifs, directly interacting with DNA or RNA. Despite their designation, CSP are present under physiological conditions and, as other stress-related proteins, have been linked to osmotic, oxidative, starvation and acid stress (Keto-Timonen et al., 2016). The *B. microti cspA* gene was identified as a promising candidate in resistance to pH 4.5 because of its higher expression under acid conditions at both time points with a RNA-Seq-based log2-fold change > 3.0, and its pseudogene character in *B. suis* due to a frameshift mutation.

**FIGURE 6:**
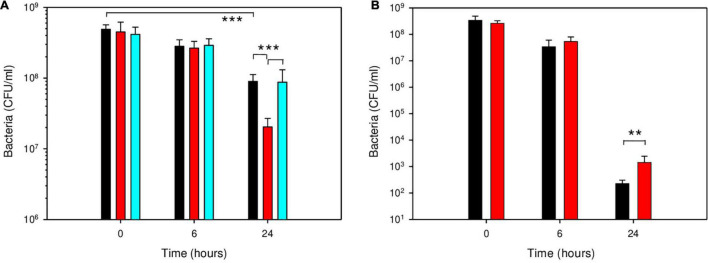
Participation of CspA in survival of *B. microti* **(A)** and *B. suis* **(B)** in GMM at pH 4.5. Survival of *B. microti* wild-type strain (black bars), *B. microti* Δ*cspA* (red bars), *B. microti* Δ*cspA* complemented with pBBR1-*cspA* (cyan bars), and of *B. suis* wild-type with pBBR1-AMP (black bars) or complemented with pBBR1-AMP-*cspA* (red bars) containing functional *cspA* of *B. microti*. Values are shown as means of 7 experiments ± SD. **: *P* < 0.01; ***: *P* < 0.001.

Deletion of *cspA* in *B. microti* reduced its survival in GMM at pH 4.5 approximately 4.5-fold at 24 h (Figure 6A). No effect was noticeable at the earlier time point of 6 h, explaining the lack of phenotype in the macrophage model of infection. Complementation of the *B. microti* Δ*cspA* strain with the native gene cloned in vector pBBR1MCS restored a level of resistance not significantly different from that of the wild-type, confirming a role of CspA in acid stress resistance at pH 4.5. To investigate if the pseudogene character of *cspA* in *B. suis* 1330 was related to the increased acid sensitivity of the classical species after 24 h at pH 4.5, *B. suis* was complemented with the intact gene from *B. microti* on the replicative plasmid pBBR1-AMP, and survival was compared to that of the wild-type. Survival of *B. suis* expressing *cspA* of *B. microti* was more than 6-fold increased (*P* < 0.01) at pH 4.5 after 24 h (Figure 6B). Although this gain of viability under these environmental conditions correlated well with the degree of loss of survival of the *B. microti* Δ*cspA* mutant strain as compared to the wild-type, general impact on survival of both species remained limited: CspA is obviously only one factor amongst others in acid resistance of *B. microti*. As expected, the causes of increased survival of *B. microti* observed in GMM at pH 4.5 are multifactorial, involving genes inactive in *B. suis* as described above and, most likely, genes differentially regulated in both species.

**FIGURE 7:**
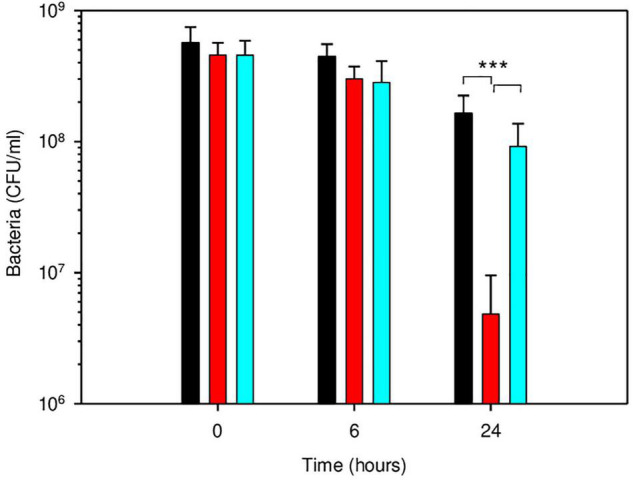
Participation of Dps in survival of *B. microti* in GMM at pH 4.5. Survival of *B. microti* wild-type strain (black bars), *B. microti* Δ*dps* (red bars), and of *B. microti* Δ*dps* complemented with pBBR1-*dps* (cyan bars). Values are shown as means of 6 experiments ± SD. ***: *P* < 0.005.

Besides CspA, the nucleoid-associated protein Dps played an important role in *B. microti* acid resistance. In *E. coli*, it participates in resistance to oxidative, UV, γ-radiation, metal ion toxicity and acid stress, and variations of nucleoid conformation have an important influence on bacterial physiology (Calhoun and Kwon, 2011). *Brucella microti dps* (BMI_I2170) was chosen for mutagenesis because of the potential biological roles of the encoded protein and its specific, higher differential expression at both time points, with a RNA-Seq-based log2-fold change > 2.0, validated by RT-qPCR (Supplementary Table 4). At 24 h, inactivation of *dps* in *B. microti* resulted in a strong reduction of survival in GMM at pH 4.5, with an approximately 34-fold decrease in the number of viable bacteria, when compared to *B. microti* wild-type (Figure 7). Complementation of the *B. microti* Δ*dps* mutant with the intact *dps* gene expressed in plasmid pBBR1MCS restored wild-type survival, confirming Dps participation in acid stress resistance at pH 4.5. As for the Δ*cspA* mutant, no effect was measurable after 6 h of exposure to pH 4.5 (Figure 7).

The Dps protein has three intrinsic properties that determine its important physiological roles: DNA-binding, resulting in DNA compaction and crystallization; iron sequestration; and ferroxidase activity, responsible for resistance to oxidative stress and protecting the DNA against ROIs. These properties play a central role in iron and hydrogen peroxide detoxification and in acid resistance. As the pH drops, interactions of Dps with DNA may become stronger, and this ability to aggregate DNA at acid pH, results in increased protection (Jeong et al., 2008). In *B. suis*, concentration of Dps increases under extreme nutrient starvation (Al Dahouk et al., 2013), evidencing together with the here-described results the important role of Dps in countering harmful environmental conditions. Moreover, *dps* expression is reported to be controlled by RpoE (Kim et al., 2014), fitting to the *B. microti*-specific increased transcription of sigma factors in our RNA-Seq study. Dps has also been detected in culture supernatants and in outer membrane vesicles (OMVs) produced by *Brucella*, alongside with other oxidative stress-related factors (Avila-Calderon et al., 2012; Lee et al., 2014), of which GroES also showed higher expression in *B. microti* at pH 4.5. This may suggest potential extracellular and pathogenesis-related functions, since both culture supernatants and OMVs have been reported to induce cytotoxic and immunogenic responses in infection models. Therefore, a possible role of Dps in pathogenesis of *Brucella* cannot be excluded and further studies would be of interest, as Dps has been linked to virulence in other pathogens (Martinez-Sanguiné et al., 2021).

In conclusion, the involvement of CspA and Dps in acid stress resistance in GMM at pH 4.5 after 24 h of exposure and the lack of phenotype in a cellular model of infection indicated that these genes may play a role in resistance of the atypical species *B. microti* to out-of-the-host conditions, such as strongly acidified soils. The lack of CspA in *B. suis* due to pseudogenization contributed to increased acid sensitivity *in vitro* but did not affect the capacity of intracellular replication of the pathogen, confirming that this gene has become dispensable in the course of adaptation to the host organism.

As mentioned earlier, neither Δ*cspA* nor Δ*dps* of *B. microti* showed phenotypes different from that of the wild-type strain in the J774 murine macrophage model of infection. A likely explanation is, that the duration of exposure to pH 4.5 in the BCV lasts only for a few hours, too short for an impact on viability of the mutant strain. Redundancy of gene functions and/or of acid stress-protective systems is also conceivable. An additional factor to be taken into consideration resides in the differences between the *in vitro* acid stress models and the intramacrophagic vacuole. GMM at pH 4.5 mimics the low nutrient and acidic environment of the phagocytic vacuole, but variability of certain parameters such as oxygen and metal ion concentrations is not considered. During the maturation process of the *Brucella* vacuole, it is likely that adaptation to acid stress is a dynamic process with variable expression profiles corresponding to the momentary conditions, and our data reflect the initial stage of this response, prone to change over time.

### *Brucella* Cell Morphology Is Affected by Acidic pH 4.5 in Gerhardt’s Minimal Medium

**FIGURE 8:**
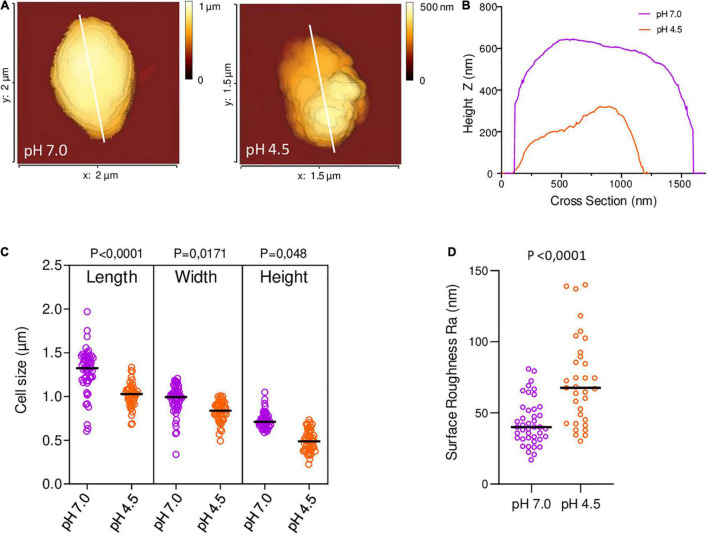
Morphological characterization of *B. microti* CCM4915 in GMM pH 7.0 and pH 4.5 by Atomic Force Microscopy (AFM). **(A)** AFM topographic images of *B. microti* at both pH values. **(B)** Topographical profile plots measured along the longitudinal axis of the cells, indicated by solid lines in **(A)**. **(C)** Length, width and height measurements of bacteria at pH 7.0 (*n* = 46) and pH 4.5 (*n* = 53). **(D)** Average roughness R_*a*_ of the bacteria at pH 7.0 (*n* = 42) and pH 4.5 (*n* = 33), based on recorded surface roughness. Cell height analysis was carried out using the height (measured) channel of the QI mode, which corresponds to the height at 80% of the setpoint force determined on the reference force-distance curve. Height was calculated as the topographical maximal central height on each cell using the section tool of the analysis software. The average roughness (Ra) was calculated on the z channel values using a 400 × 400 nm area for each cell. Statistical differences were analyzed by *t*-test and yielded P values < 0.05, as indicated for panels **(C)** and **(D)**.

The impact of GMM pH 4.5 on *Brucella* cell morphology has been studied for *B. microti* using atomic force microscopy (AFM). At acidic pH, bacteria are significantly shorter and “flatter” than at pH 7, resulting in volume reduction (Figure 8). In addition, cell surface roughness is considerably increased at low pH, indicating potential acid-mediated damage on proteins and/or LPS (Figure 8). Bacterial shape is dependent on peptidoglycan and on the bacterial cytoskeleton, whereby cellular localization of components of the latter is controlled by the membrane potential (Jones et al., 2001; Strahl and Hamoen, 2010). As a consequence, acid stress, altering this potential, may induce the morphological changes observed. Furthermore, due to the interconnection of osmoregulation and pH homeostasis, a sudden modification in one of the systems may create an imbalance, affecting also the other and possibly bacterial morphology.

## General Conclusion

**FIGURE 9:**
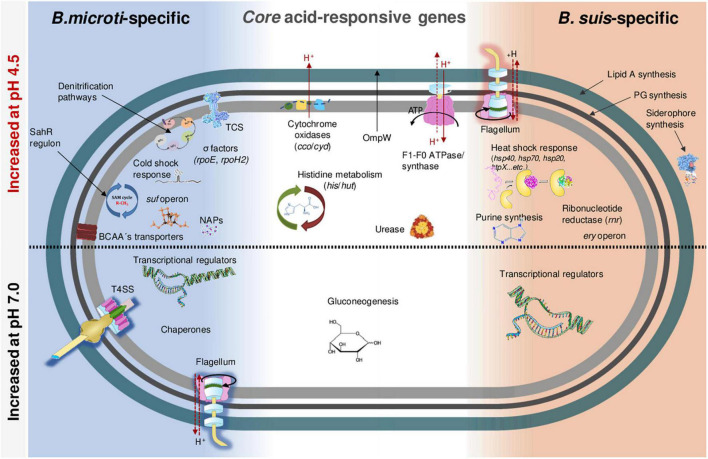
Schematic representation of the highlighted common and species-specific responses for both *Brucella* species. For the experimental time points chosen, genes with higher expression at pH 4.5 are shown in the upper half, those with higher expression at pH 7 in the lower half of the scheme.

An overview of major results presented above is given in Figure 9, which illustrates the highlighted common and species-specific responses for both *Brucella* species.

Most central elements in the core response to pH 4.5 during the early phase of acid stress are the increased rates of expression of genes for cytochrome oxidases, the F_1_F_0_-ATPase/ATP-synthase, and the histidine metabolism together with the urease system. These systems have in common proton neutralization or extrusion from the cytosol, maintaining physiological intracellular pH values. In addition, species-specific mechanisms were identified that resulted in the conclusion that both species faced acid stress in distinct ways. At pH 4.5, *B. microti* increased expression of: (1) key elements of the general stress response; (2) genes of all denitrification steps, favoring rapid adaptation to acid-induced low oxygen conditions; (3) methionine metabolism genes, involved in DNA-methylation and in sulfur metabolism participating in redox homeostasis through biosynthesis of thiol antioxidants; (4) genes involved in Fe-S-cluster assembly, countering redox-state disturbance; (5) cold shock proteins, possibly acting as RNA chaperones; (6) nucleoid-associated protein Dps, playing a protective role in stress resistance by increased DNA aggregation. Altogether, these observations revealed rapid physiological adaptation of *B. microti* to acid stress, anticipating potential damage to cellular components and critical energy conditions.

On the other hand, at pH 4.5, *B. suis* increased expression of genes participating in: (1) purine and ribonucleotide-diphosphate reductase, (2) peptidoglycan, and (3) lipid A biosynthesis, indirectly suggesting increased levels of structural damages of the DNA, cell wall and outer membrane; (4) erythritol metabolism, possibly reflecting specific metabolic adaptation to the within-the-host life; (5) siderophore biosynthesis, indicating an important need for iron during acid stress; (6) flagella assembly, potentially contributing to proton export; (7) heat shock response, suggesting significantly increased acid stress-mediated protein misfolding or damaging in *B. suis*. The classical species therefore set up an array of repair strategies aiming at countering the symptoms rather than the origins of acid stress, resulting in subsequent loss of viability.

Remarkable was the identification of 15 acid stress-induced genes, all pseudogenes exclusively in *B. suis*. This loss of functionality during the host adaptation process most likely participated in reduced *B. suis* acid stress resistance when compared to the more ancestral and free-living species *B. microti*, as confirmed for the cold shock protein CspA. Dps, on the other hand, is an example for a factor whose pH-dependent regulation of expression in *B. microti* coincides with increased resistance of this species to pH 4.5. *B. microti* mutants with reduced survival at pH 4.5 in GMM showed a phenotype only after more than 6 h of exposure, explaining why none of the mutants tested were affected in the macrophage, where the duration of BCV acidification is limited. This observation was also coherent with the host adaptation-related accumulation of pseudogenes in *B. suis*, not impacting intracellular survival.

In conclusion, our work supported the hypothesis that increased acid stress resistance of *B. microti* was based on selective pressure for the maintenance of functionality of critical genes, and on specific differential gene expression, resulting in rapid adaptation.

## Data Availability Statement

The datasets presented in this study can be found in online repositories. The names of the repository/repositories and accession number(s) can be found below: https://www.ncbi.nlm.nih.gov/, PRJNA644280.

## Author Contributions

SK, AO, and SA designed the study. JG-G, SO-B, SL, EO-E, LF, SK, and AO carried out the experiments and/or performed analysis of the data. SK, AO, JG-G, and SA were involved in drafting the manuscript. All authors read and approved the final manuscript.

## Conflict of Interest

The authors declare that the research was conducted in the absence of any commercial or financial relationships that could be construed as a potential conflict of interest.

## Publisher’s Note

All claims expressed in this article are solely those of the authors and do not necessarily represent those of their affiliated organizations, or those of the publisher, the editors and the reviewers. Any product that may be evaluated in this article, or claim that may be made by its manufacturer, is not guaranteed or endorsed by the publisher.

## Footnotes

1
https://www.genoscope.cns.fr/agc/microscope/mage/viewer.php

2
http://www.phidias.us/victors/index.php

3
http://www.mgc.ac.cn/VFs/main.htm

4
https://www.patricbrc.org/

5
https://www.genome.jp/kegg/pathway.html

6
https://www.uniprot.org/uniprot/Q2YMS6
