# Supplementary material for: Comparative Genome-Wide Transcriptome Analysis of Brucella suis and Brucella microti Under Acid Stress at pH 4.5: Cold Shock Protein CspA and Dps Are Associated With Acid Resistance of B. microti
Source: Front Microbiol. 2021 Dec 13;12:794535. doi: 10.3389/fmicb.2021.794535 (PMC8710502; doi:10.3389/fmicb.2021.794535)
Supplement: Supplementary file 7 [file Image_1.PDF]

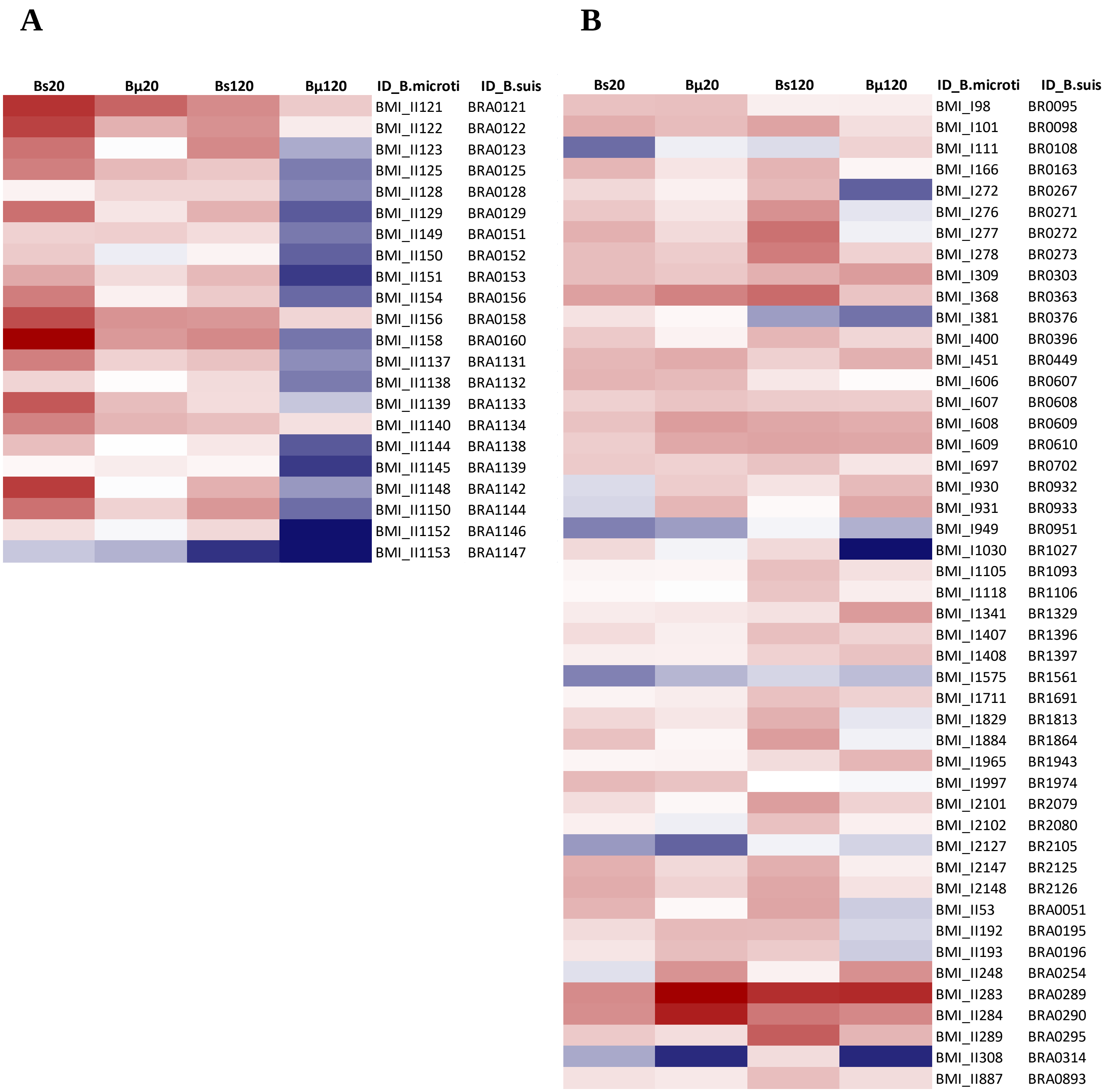

Supplementary Figure 1. Heatmaps of genes associated to (A) COG N (cell motility) or (B) COG O (Posttranslational modification/protein turnover/chaperones) and differentially expressed in *B. suis* and/or *B. microti* at pH 4.5/pH 7. The columns represent for each gene the pH 4.5/pH 7.0 expression ratios for Bs20 (*B. suis* at 20 min.), Bμ20 (*B. microti* at 20 min.), Bs120 (*B. suis* at 120 min.) and Bμ120 (*B. microti* at 120 min.). Heatmaps were created using the Microsoft® Excel® 2019 color scale tool. Red: increased expression at pH 4.5; blue: increased expression at pH 7.0.
